# The Effects of Prenatal Alcohol Exposure

**Published:** 1997

**Authors:** Cynthia Larkby, Nancy Day

**Affiliations:** Cynthia Larkby, M.S.W., is a doctoral candidate and Nancy Day, M.P.H., Ph.D., is a professor of psychiatry, epidemiology, and pediatrics and director of the Program in Epidemiology at Western Psychiatric Institute and Clinic, University of Pittsburgh School of Medicine, Pittsburgh, Pennsylvania

**Keywords:** gestation, prenatal alcohol exposure, fetal alcohol effects, fetal alcohol syndrome, teratogens, congenital anomaly, fetal development, disorder definition, epidemiology, amount of AOD use, growth retardation, morphology, CNS function, mental retardation, cognitive development, demographic characteristics, psychosocial environment, literature review

## Abstract

Exposure to alcohol during gestation can cause persistent abnormalities in physical and cognitive development. Children who meet the clinical definition of fetal alcohol syndrome (FAS) are small for their age, exhibit characteristic facial anomalies, and demonstrate deficits in central nervous system development. Alcohol effects in children with prenatal exposure, but not FAS, are similar, although of smaller magnitude and not necessarily present in all three systems. The degree to which a person is affected by prenatal alcohol exposure depends on the amount, timing, and duration of the mother’s alcohol consumption during pregnancy as well as maternal characteristics (e.g., age and comorbid psychiatric disorders) and environmental factors (e.g., socioeconomic status and family problems). Some exposure-related effects, such as growth deficits, are directly related to the amount of alcohol consumed, however, so that even a small amount of alcohol may affect child development. Therefore, the best policy continues to be abstinence during pregnancy.

Pregnant alcoholic women risk the health of their offspring in multiple ways: (1) Exposure to alcohol during gestation may lead to fetal alcohol syndrome (FAS) or fetal alcohol effects; (2) the physical consequences of alcoholism in the mother (e.g., falls or malnutrition) may independently affect the developing fetus; (3) genetic vulnerability to alcoholism in the fetus may increase the effects of prenatal exposure; and (4) the lifestyle of an alcoholic parent may lead to negative consequences for the fetus, the pregnancy, and the developing child. This article addresses the first of these issues—the effects of exposure to alcohol during gestation—in detail. However, any or all of the other issues listed (i.e., concomitant genetic background and the physical and lifestyle deficits that accompany alcoholism) may exacerbate the adverse effects of prenatal alcohol exposure.

As a teratogen, alcohol is capable of directly inducing developmental abnormalities in a fetus. Alcohol use during pregnancy is one of the most common known causes of preventable birth defects, and its results can persist as long-term deficits in physical and cognitive growth and development.

The dangers of fetal alcohol exposure, initially identified in the late 1960’s, are entirely preventable if women abstain from drinking during pregnancy. Given this fact, in 1981 the U.S. Surgeon General issued the first health advisory recommending that women who are pregnant or planning a pregnancy should not drink alcohol, and this advisory was repeated in 1990 and 1995.

## FAS Definition and Diagnosis

At the extreme end of the spectrum of prenatal exposure effects, FAS is a clinical diagnosis applied to children who have been exposed to alcohol during gestation and exhibit deficits in growth, physical structure (i.e., morphology), and the central nervous system (CNS). To meet the clinical case definition, the child must have symptoms in each of the following three categories: (1) growth deficiency in both the prenatal and postnatal periods; (2) abnormalities in facial and skull structure, including small eye openings (i.e., short palpebral fissures), alterations in nose and forehead structure, an absent or elongated groove between the upper lip and nose (i.e., philtrum), a thin upper lip, a flattened midface, and underdevelopment of the upper or lower jaw; and (3) CNS deficits, such as mental retardation and behavioral problems (Sokol and Clarren 1989). Of these symptoms, the facial abnormalities are the most characteristic of FAS, whereas the CNS anomalies have the most significant effect on overall development. Separately, each of these features is defined as an alcohol-related birth defect (ARBD) or a fetal alcohol effect.

The features associated with FAS may change with age, complicating the diagnosis. Before age 2, CNS dysfunction is difficult to assess, and the classic facial abnormalities (see figure) may not be clearly evident. At older ages, growth deficits are offset by the adolescent growth spurt as well as normal changes in facial length and width associated with maturation. Because of these changes, growth deficits and facial features become less apparent after puberty, and without prepubertal photographs and reliable growth records, FAS may be difficult to diagnose in adolescents or adults.

**Figure:**
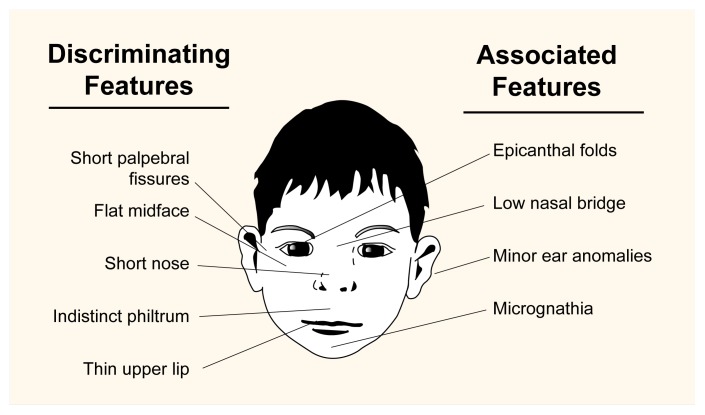
Facial features particularly characteristic of a child with fetal alcohol syndrome (FAS). Discriminating features (i.e., those considered definitive signs of FAS) are shown on the left side of the illustration; characteristics listed on the right side are associated with FAS but are not sufficient to determine the presence of the syndrome. Microencephaly (small head circumference) is not a facial feature per se, but a central nervous system characteristic. (Palpebral fissures = eye openings; philtrum = groove between nose and upper lip; epicanthal folds = skin folds covering inner corner of the eye; micrognathia = abnormal smallness of the jaws.) SOURCE: Streissguth and Little 1994.

Accurately assessing fetal alcohol exposure may prove even more difficult. To obtain correct information regarding the quantity of alcohol consumed as well as the timing and duration of alcohol use during pregnancy, clinicians and researchers need reliable methods to determine alcohol exposure. Although biological tests are available to measure the amount of alcohol consumed, these tests reflect use over a very short time period and do not allow estimates of the pattern or duration of use to be made. In general, clinicians and researchers rely on the mother’s self-report of alcohol use. Self-reports may be inaccurate, however, because social pressures, fear of being labeled, and anxiety about losing custody of her child may lead a woman to underreport her alcohol consumption during pregnancy. Problems recalling the frequency and quantity of alcohol consumed also can lead to erroneous estimates. In the absence of an accurate report, clinicians and caretakers, such as foster and adoptive parents, will not know whether or to what extent the child was exposed to alcohol during gestation. Therefore, both clinicians and researchers must establish trust and rapport with all pregnant women to enable them to report their alcohol use both honestly and accurately.

Because of the difficulty in reliably ascertaining fetal alcohol exposure, a committee convened by the Institute of Medicine to study FAS recently reviewed the diagnostic criteria currently in use and recommended revisions. The committee members proposed a diagnostic classification system with five categories: (1) FAS with confirmed maternal alcohol exposure, (2) FAS without confirmed maternal alcohol exposure, (3) partial FAS with confirmed maternal alcohol exposure, (4) ARBD with a history of maternal alcohol exposure, and (5) alcohol-related neurodevelopmental disorder (ARND) with a history of maternal alcohol exposure (Stratton et al. 1996). The last two categories are used for offspring who have morphologic and neurologic alcohol-related effects, respectively, without the full features of FAS, and they may co-occur in the same individual.

## FAS Epidemiology

### Alcoholism and Alcohol Use Among Women

In a nationwide household survey conducted in 1992, 4.08 percent of the women who were interviewed met the criteria^1^ for alcohol abuse and/or dependence within the 12 months preceding the survey (Grant et al. 1994). The highest prevalence rates for alcohol abuse and/or dependence were found among women of childbearing age (9.84 percent for women ages 18 to 29 and 3.98 percent for women ages 30 to 44).

Women who are alcoholic typically experience several other problems, including comorbid medical or psychiatric disorders (e.g., depression) and social problems (e.g., unstable marriages; spouses with drinking problems; and child-care responsibilities, often as single mothers). This multiplicity of problems complicates the pregnancy of an alcoholic woman, because her fetus is exposed not only to the teratogenic effects of alcohol, but also to the negative effects of the other factors that coexist in her life. By comparing birth outcomes in upper middle class and lower class alcoholic mothers, Bingol and colleagues (1987) showed that economic or lifestyle factors play a role in the rate of FAS. They found that, although the intake of absolute alcohol was equivalent in the two groups, 2.7 percent of the upper middle class mothers had a child with FAS, compared with 40.5 percent of the lower class mothers.

The problem of alcohol exposure during pregnancy is not limited to alcoholic women, however. A majority of women drink, as evidenced by a national household survey (Hilton 1991) in which 64 percent of the women surveyed were drinkers (i.e., drank at least once a year), 4 percent reported daily drinking, 6 percent drank five or more drinks on occasion at least weekly, and 1 percent reported drinking enough to feel drunk at least weekly. Age, race, social class, and occupation all predicted drinking patterns among women. For example, younger women were most likely to drink, and white women drank more than their black or Hispanic counterparts. Drinkers had a higher education level and income than nondrinkers and were more likely to work full time outside the home. These same four characteristics also described women who were heavy drinkers.^2^

Despite the fact that women’s drinking rates have remained relatively constant since the 1960’s (Hilton 1991), the rate of drinking during pregnancy appears to be increasing. For example, the Behavioral Risk Factor Surveillance System reported that among pregnant women, the rate of drinking increased from 12.4 percent in 1991 to 16.3 percent in 1995 and that the rate of frequent drinking^3^ was four times higher in 1995 than in 1991, increasing from 0.8 percent in 1991 to 3.5 percent in 1995 (Centers for Disease Control and Prevention 1997).

### Prevalence of FAS

Abel and Sokol (1987) estimated that approximately 6 percent of the offspring of alcoholic women have FAS, although for offspring born after an FAS sibling, the risk is very high (70 percent) (Abel 1988). The high probability that all subsequent offspring will be affected after an initial case means that some consequence of chronic alcoholism in addition to alcohol exposure must lead to the higher rate of FAS among offspring born later. Even in the absence of full-blown FAS, babies of alcoholic women have a higher rate of the separate characteristics of FAS.

Among the general population, a national surveillance program known as the Birth Defects Monitoring Program reported a rate of 5.2 FAS cases per 10,000 live births (Cordero et al. 1994). Similarly, in a recent summary of findings from prospective studies, Abel and Sokol (1991) estimated the overall rate of FAS to be 3.3 cases per 10,000 live births. The rate of FAS is likely to be considerably underestimated, however, because of the difficulty in making the diagnosis and the reluctance of clinicians to label children and mothers. For example, Little and colleagues (1990) studied the outcomes of 40 pregnancies of 38 women whose alcohol abuse was noted in their medical records. Although one-half of the 34 liveborn infants demonstrated poor postnatal growth and development and 6 neonates met the diagnostic criteria for FAS, none of the infants was diagnosed as having FAS.

## Relationship of Exposure and Effects

Fetal development is a sequential, multistaged process. To determine the effects of prenatal exposure on child development, factors such as the timing, dose, and pattern of alcohol exposure must be considered, because growth, morphologic abnormalities, and CNS deficits occur at different points during gestation. Major morphologic abnormalities result from exposure early in pregnancy, growth is most affected by late exposure, and CNS deficits occur throughout gestation. Thus, offspring who are exposed to alcohol throughout pregnancy will not have the same outcome as offspring who are exposed only during early pregnancy or only at specific times during pregnancy.

Identifying the nature of the relationships between prenatal alcohol exposure and outcome is also important for research and clinical reasons. Exposure to a toxin may affect fetal outcome in two ways: The effect may be directly related to the amount of exposure (i.e., a linear relationship), or exposure may be problematic only above a certain level (i.e., a threshold relationship). A linear relationship between alcohol exposure and child development means that no “safe” level of drinking during pregnancy exists, because even a small amount of alcohol could produce an effect. In contrast, the threshold model implies that a “safe” level of drinking does exist, below which negative effects do not occur. Data from studies to date demonstrate that the relationship between alcohol exposure and outcome varies depending on the type of outcome under consideration, however. For example, reports from the animal (Schenker et al. 1990) and human literature (Sampson et al. 1989; Goldschmidt et al. 1996) support a threshold relationship between prenatal alcohol exposure and CNS development, whereas the data on physical growth indicate that the effect of gestational exposure to alcohol is linear (Day et al. 1994). Therefore, no “safe” level of consumption exists, and the best policy for women continues to be abstinence during pregnancy to avoid any negative effects on their offspring.

## Specific Effects of Prenatal Exposure

People with FAS demonstrate growth deficits, morphologic abnormalities, mental retardation, and behavioral difficulties. In a systematic followup study, Streissguth and colleagues (1991) examined 61 subjects identified as having FAS or fetal alcohol effects to determine adolescent and adult manifestations of the syndrome. The study results give an overview of the long-term impact of prenatal alcohol exposure. At adolescence and adulthood, the subjects were short in stature and small in head circumference; they also exhibited a high rate of abnormal facial features, although these characteristics were not as pronounced as they had been at younger ages. The subjects’ IQ scores ranged from 20 to 105 with a mean of 68. Six percent of the subjects took regular school classes, but the remainder of the study participants were unable to achieve this schooling level or maintain regular outside employment. In addition, the subjects demonstrated poor concentration and attention; an inability to live independently in adulthood; stubbornness; social withdrawal; and conduct problems, such as lying, cheating, and stealing. Their characteristics and long-term outcome predict the expected outcome for people who were prenatally exposed to alcohol but do not have the full syndrome. At lower levels of exposure, a subset of fetal alcohol effects, rather than the full extent of FAS features, is most likely to occur.

The following sections describe the effects of prenatal alcohol exposure on growth, morphology, and CNS development in turn. Many of the examples are taken from the Maternal Health Practices and Child Development (MHPCD) project, a longitudinal study of the long-term effects of prenatal alcohol exposure. In this study, researchers recruited adult women in their fourth month of pregnancy from a prenatal clinic. All women who consumed an average of three or more drinks per week during their first trimester, plus a random sample of one-third of the women who drank alcohol less often, were selected as study subjects. In general, alcohol use during pregnancy was light to moderate among the women participating in the study, although subjects who represented the entire spectrum of use were included in the sample.

### Growth Deficits

Children with FAS are small for their age (Streissguth et al. 1991)—indeed, such smallness is one of the criteria for diagnosis, although growth deficits also are found among children who were exposed to alcohol during pregnancy but do not fulfill the full criteria for FAS. As noted previously, however, growth retardation is somewhat ameliorated at puberty. In the MHPCD project, these growth deficits are symmetrical, affecting height, weight, and head circumference to the same degree, and remain significant through age 10. The relationship between prenatal exposure and growth deficits is linear (i.e., the greater the prenatal alcohol exposure, the more pronounced the effect on postnatal growth). Smith and colleagues (1986) also found that the duration of exposure, in addition to amount, affected birth weight.

Postnatal environment and maternal characteristics influence the relationship between prenatal alcohol exposure and growth, however. Whereas studies of disadvantaged populations (Coles et al. 1991; Day et al. 1994; Jacobson et al. 1994*b*; Russell 1991) have found that prenatal alcohol exposure continues to affect growth at followup, studies of more advantaged cohorts (Barr et al. 1984; Fried and O’Connell 1987; O’Connor et al. 1986) have found that growth deficits are not maintained as the children get older. Another study (Jacobson et al. 1994*a*) found that alcohol exposure was associated with decreased birth weight, length, and head circumference, although only among infants of women over age 30. Thus, postnatal environment and maternal characteristics apparently exacerbate the effects of prenatal alcohol exposure.

### Morphologic Abnormalities

Another criterion of FAS is the presence of the specific group of facial anomalies mentioned previously (i.e., short palpebral fissures, a flattened nasal bridge, an absent or elongated philtrum, and a thin upper lip). From embryological studies, investigators know that these morphologic abnormalities occur when the midline of the face is formed during the first trimester. A significant correlation between first-trimester alcohol exposure and the rate of these physical anomalies was found in the MHPCD project (Day et al. 1990). As noted in other studies of FAS, however, the relationship between prenatal alcohol exposure and the characteristic facial features associated with FAS diminished as the children matured.

### CNS Deficits

Both animal and human studies have demonstrated that brain structures, including the hippocampus, frontal lobes, corpus callosum, and basal ganglia, are important sites of alcohol’s action on the fetal brain (Clarren et al. 1978; Coulter et al. 1993; Mattson et al. 1992, 1996*b*; Pfeiffer et al. 1979; Riley et al. 1995; Shapiro et al. 1984; Wisniewski et al. 1983). Indeed, researchers have documented anomalies of brain structure and function among children with FAS. Evidence of CNS deficits in FAS children also appears in their tendency to have delayed motor and speech development and speech and hearing impairments (Steinhausen et al. 1982; Church and Gerkin 1988). In prenatally exposed children who do not have FAS, researchers have identified neurologic effects at birth that reflect abnormalities in sleep patterns (Scher et al. 1988) and in the newborn’s ability to respond and adapt, as measured by the Brazelton Neonatal Behavioral Assessment Scale (Coles et al. 1985; Streissguth et al. 1983).

One way to gauge CNS functioning is to use neuropsychological measures designed to assess brain functioning. Using such measures, Mattson and colleagues (1996*a*) found that 5- to 16-year-old children with FAS had significant verbal learning and memory deficits. Similarly, Kodituwakku and colleagues (1995) reported memory deficits in 13-year-old children with FAS, and Coles and colleagues (1997) found that children with FAS had deficits in problem-solving, information processing and storage, and visual and spatial skills.

The neuropsychological findings are similar for children who were exposed to alcohol during gestation but do not have FAS. Jacobson and colleagues (1993) reported that prenatally exposed 13-month-old infants were slower or less efficient at information processing. Such deficits apparently persist: In a study by Streissguth and colleagues (1994), 14-year-old children who had been prenatally exposed to alcohol had difficulty performing tasks that required processing information in order to make complex decisions. Researchers also have found that prenatally exposed children have particular difficulty in mathematical tasks (Kopera-Frye et al. 1996). In another study, Coles and colleagues (1991) compared the cognitive performance of children whose mothers drank an average of 11.8 ounces of absolute alcohol (i.e., approximately 24 drinks) per week throughout pregnancy and children whose mothers stopped drinking in the second trimester or did not drink at all during pregnancy. The researchers found that the children exposed throughout gestation performed more poorly than children in the other two groups, exhibiting deficits in short-term memory and encoding (i.e., sequential processing) and overall mental processing at an average age of 5 years and 10 months.

People with FAS often are mentally retarded, although the degree of deficit varies (Landesman-Dwyer 1982; Streissguth et al. 1991). Streissguth and colleagues (1996) reported that the IQ scores of FAS patients ranged from 29 (severely retarded) to 120 (high average). Like other exposure-related effects, the impact of prenatal alcohol exposure on cognitive development demonstrates a continuum. Although study results are not completely consistent, alcohol exposure is related to decreased cognitive abilities even at lower levels of exposure. For example, Streissguth and colleagues (1989*a*) reported that the daily consumption of 1½ ounces of absolute alcohol (i.e., approximately three drinks) was associated with an average decrease of 5 points in the child’s IQ score at age 4. At age 7½, children exposed to more than 1 ounce of absolute alcohol (i.e., approximately two drinks) per day scored an average of 7 IQ points lower compared with children not exposed to this amount (Streissguth et al. 1990).

People with FAS commonly exhibit behavioral problems as well (Majewski 1978*a*; Majewski 1978*b*; Olegard et al. 1979; Shaywitz et al. 1980; Steinhausen et al. 1982; Streissguth et al. 1991). These problems can include poor concentration and attention, lack of independent living skills, stubbornness, and social withdrawal. In addition, children with FAS exhibit higher rates of conduct problems (e.g., lying, cheating, and stealing). Streissguth (1993) reported that as children with FAS mature, they demonstrate poor socialization and communication skills and commonly experience problems with alcohol and drug abuse and antisocial behavior.

Behavior problems also have been reported among offspring prenatally exposed to alcohol but without FAS. In one study, 4-year-old children whose mothers drank one to five drinks per day during pregnancy were less attentive and more active when observed in the home, compared with children of control mothers who drank less (Landesman-Dwyer 1982). At age 7½, the children were less attentive and took a longer time to react to a stimulus on a Continuous Performance Task (Streissguth et al. 1986). In children ages 7 (Streissguth et al. 1989*b*) and 14 (Streissguth et al. 1994), researchers demonstrated the effects of prenatal exposure to alcohol on both attention and memory. These effects were linear (i.e., the extent of the effect was directly correlated with the amount of alcohol exposure), implying that no “safe” threshold of alcohol exposure exists.

In the MHPCD project, mothers and teachers described children ages 3, 6, and 10 who were prenatally exposed to alcohol as showing increased activity and poorer attention, as well as social problems, anxiety, and depression (Day 1997). Brown and colleagues (1991) noted that children who had been exposed to alcohol throughout pregnancy showed deficits in their ability to sustain attention, and their teachers reported that they had problems with both attention and behavior in school.

Clinical studies provide further evidence of the neurobehavioral consequences of prenatal alcohol exposure. Such studies have reported that people with FAS experience trouble in school and maintaining jobs, a likely compound of their lower IQ scores, neuropsychological deficits, and behavior problems. Even among children and adults who do not have FAS, lower academic achievement is significantly related to prenatal alcohol exposure (Coles et al. 1991; Streissguth et al. 1990). An analysis of the outcomes among 6-year-olds in the MHPCD project, for example, demonstrated effects of second-trimester alcohol exposure on reading, spelling, and mathematics skills (Goldschmidt et al. 1996). Coles and colleagues (1991) also found that children who were exposed in early pregnancy performed more poorly in mathematics and reading than their peers who had not been exposed.

## Summary and Conclusions

In summary, cases of FAS are characterized by abnormalities in growth, morphology, and CNS development. Among exposed offspring who do not have FAS, deficits are seen in the same pattern, although they may be of smaller magnitude and do not affect all three systems in each person. Therefore, the effects of prenatal alcohol exposure range over a continuum from fully developed FAS to the milder constellation of fetal alcohol effects.

Studies show that the effects of prenatal alcohol exposure can be influenced by maternal characteristics, such as age and comorbid psychiatric disorders, or by factors in the postnatal environment such as socioeconomic status and family problems. Thus, the interaction between a vulnerable child and a disadvantaged environment compounds the negative outcomes.

There is a pressing need to understand the broad picture of the combined effects of alcohol exposure, poverty, and lifestyle on the developing fetus. Each of these risk factors for poor pregnancy outcome must be considered in evaluating the effects of prenatal alcohol use, because it is unclear whether alcohol effects occur independently or in interaction with risk factors such as an impoverished social environment.

Given that alcohol is a teratogen, an appropriate goal would be to eliminate drinking during pregnancy. This means finding effective methods to help women who are alcoholic abstain during pregnancy and to motivate other drinking women to abstain from levels of alcohol consumption that would be insignificant outside of pregnancy. Clinicians need to ask pregnant women about their alcohol consumption, even at a “social drinking” level, and have appropriate tools available to intervene when necessary. Little research has been conducted on the effectiveness of alcohol treatment during pregnancy or the treatment of pregnant alcoholics, although these women are at greatest risk for having an FAS child.

## Footnotes

1 The criteria used in this study corresponded to the criteria for alcohol abuse and dependence listed in the American Psychiatric Association’s *Diagnostic and Statistical Manual of Mental Disorders, Fourth Edition*. In general, the terms “alcoholic” and “alcoholism,” as used in this article, encompass both diagnoses.

2 In this study, “heavy drinkers” were defined as people who consumed eight or more drinks per day as often as three times per week.

3 “Frequent drinking” was defined in this study as consuming seven or more drinks per week or five or more drinks on an occasion in the past month.
